# LINNAEUS: A species name identification system for biomedical literature

**DOI:** 10.1186/1471-2105-11-85

**Published:** 2010-02-11

**Authors:** Martin Gerner, Goran Nenadic, Casey M Bergman

**Affiliations:** 1 Faculty of Life Sciences, University of Manchester, Manchester, M13 9PT, UK; 2 School of Computer Science, University of Manchester, Manchester, M13 9PL, UK

## Abstract

**Background:**

The task of recognizing and identifying species names in biomedical literature has recently been regarded as critical for a number of applications in text and data mining, including gene name recognition, species-specific document retrieval, and semantic enrichment of biomedical articles.

**Results:**

In this paper we describe an open-source species name recognition and normalization software system, LINNAEUS, and evaluate its performance relative to several automatically generated biomedical corpora, as well as a novel corpus of full-text documents manually annotated for species mentions. LINNAEUS uses a dictionary-based approach (implemented as an efficient deterministic finite-state automaton) to identify species names and a set of heuristics to resolve ambiguous mentions. When compared against our manually annotated corpus, LINNAEUS performs with 94% recall and 97% precision at the mention level, and 98% recall and 90% precision at the document level. Our system successfully solves the problem of disambiguating uncertain species mentions, with 97% of all mentions in PubMed Central full-text documents resolved to unambiguous NCBI taxonomy identifiers.

**Conclusions:**

LINNAEUS is an open source, stand-alone software system capable of recognizing and normalizing species name mentions with speed and accuracy, and can therefore be integrated into a range of bioinformatics and text-mining applications. The software and manually annotated corpus can be downloaded freely at http://linnaeus.sourceforge.net/.

## Background

The amount of biomedical literature available to researchers is growing exponentially, with over 18 million article entries now available in MEDLINE [1] and over a million full-text articles freely available in PubMed Central (PMC) [2]. This vast information resource presents opportunities for automatically extracting structured information from these biomedical articles through the use of text mining. A wide variety of biomedical text-mining tasks are currently being pursued (reviewed in [3,4]), such as entity recognition (*e.g. *finding mentions of genes, proteins, diseases) and extraction of molecular relationships (*e.g. *protein-protein interactions). Many of these systems are constructed in a modular fashion and rely on the results of other text-mining applications. For example, in order to extract the potential interactions between two proteins, the proteins themselves first need to be correctly detected and identified.

One application that could facilitate the construction of more complex text-mining systems is accurate species name recognition and normalization software (*i.e. *software that can tag species names in text and map them to unique database identifiers). For example, if the species and locations of species mentions discussed in a document were known, it could provide important information to guide the recognition, normalization and disambiguation of other entities like genes [5-7], since genes are often mentioned together with their host species. In recent text-mining challenges such as the identification of protein-protein-interactions at BioCreative II [8] or bio-molecular event extraction at the BioNLP shared task [9], some groups considered species identification and normalization an essential sub-task [10]. Likewise, improved methods for identifying species names can assist pipelines that integrate biological data using species names as identifiers [11,12].

In addition to being useful for more complex text-mining and bioinformatics applications, species name recognition software would also be useful for "taxonomically intelligent information retrieval" [13]. Document search queries could be filtered on the basis of which species are mentioned in the documents [14], providing researchers more fine-grained control over literature search results. This use case provides a powerful extension to simple keyword-based PubMed searches, since all synonyms of a species would be normalized to a standard database identifier, and could therefore be retrieved by any synonym used as input. This can currently be done to some degree by specifying Medical Subject Heading (MeSH) terms when performing a PubMed query. However, MeSH-based queries have limitations since the set of MeSH tags comprises only a small subset of all species. Additionally, semantic enhancement (marking-up entities in text and hyper-linking them to external databases [15,16]) of research articles with species names could provide readers with easier access to a wealth of information about the study organism. Accurate recognition and normalization of species mentions in biological literature would also facilitate the emerging field of biodiversity informatics, which aims to develop databases of information on the description, abundance and geographic distribution of species and higher-order taxonomic units [13,17,18].

The task of identifying species names in biomedical text presents several challenges [10,13,19], including: (i) Species name ambiguity: many abbreviated species names are highly ambiguous (*e.g. *"C. elegans" is a valid abbreviation for 41 different species in the NCBI taxonomy). Ambiguity is also introduced because names can refer to different NCBI taxonomy species entries (*e.g. *"rats" can refer to either *Rattus norvegicus *or *Rattus sp.*). (ii) Homonymy with common words: some species common names are widely used in general English text (*e.g. *"Spot" for *Leiostomus xanthurus *and "Permit" for *Trachinotus falcatus*). These names introduce a large number of false positives if not properly filtered. (iii) Acronym ambiguity: species dictionaries contain acronyms for species names (*e.g. *HIV for Human immunodeficiency virus), which can refer to multiple species or other non-species entities. In fact, it has previously been shown that 81.2% of acronyms in MEDLINE have more than one expansion [20]. This presents challenges relating to identifying when an acronym refers to a species, and, if so, which species when it refers to several. (iv) Variability: while species dictionaries cover a large number of scientific names, synonyms and even some common misspellings, they cannot match human authors in variability of term usage. In some cases, authors use non-standard names when referring to species, spell names incorrectly or use incorrect case.

Despite these challenges, several attempts have been made to automate the process of species name recognition and normalization using a range of different text mining approaches. Previous efforts in species name recognition can broadly be categorized in two groups: software aiming to identify species names in legacy documents in the field of biodiversity (e.g. the Biodiversity Heritage Library [21]), and software aiming to identify species names in current biomedical literature (e.g. MEDLINE or PubMed Central). The main aim of tools profiled towards the field of biodiversity is to recognize as many species names as possible, many of which have not been recorded in existing species dictionaries. Biodiversity-oriented methods typically use rule-based approaches that rely on the structure of binomial nomenclature for species names adopted by Carl Linnaeus [22]. By taking advantage of regularity in naming conventions, these approaches do not have to be updated or re-trained as new dictionary versions are released or species names change, and can cope with the very large number of possible species names in the biodiversity literature. However, rule-based methods are often unable to identify common names (*e.g. Drosophila melanogaster *follows the typical species name structure, while "fruit fly" does not).

TaxonGrab [23] is such a rule-based tool, which consists of a number of rules based on regular expressions. Using an English-language dictionary, it finds all words that are not in the common-language dictionary, and applies rules based on character case and term order in order to determine whether a term is a species name or not. It is implemented in PHP and available under an open-source license [24]. TaxonGrab performance is high (94% recall, 96% precision) against a single 5000-page volume on bird taxonomy, but it has not been evaluated on biomedical articles. "Find all taxon names" (FAT) [25] is a more complex mention-level method related to TaxonGrab, with several additional rules aimed at increasing recall and precision. FAT reports better accuracy than TaxonGrab (>99% recall and precision) on the same evaluation set and can be accessed through the GoldenGate document mark-up system [26,27]. It is important to note, however, that the performance of these methods has not been evaluated against normalization to database identifiers.

The uBio project provides a set of modular web services for species identification [28] and automatic categorization of articles based on the species mentioned in them [11]. FindIT, part of the uBio suite, is a rule-based system aiming to perform species name recognition, aided by a range of dictionaries. After recognition, a confidence score is given for each match and, where possible, any recognized species names are mapped to uBio Namebank records. However, like TaxonGrab, FindIT is unable to recognize common names such as "human." TaxonFinder is a related method influenced by both TaxonGrab and FindIT, that brings together elements from both systems [29,30]. MapIT performs species name normalization by mapping species names to a taxonomic tree rather than directly to a database identifier. The implementation is not described in detail and no evaluation of the system is reported. Our testing of the system reveals that MapIT will map common names such as "human" to any species with a name or synonym that contains human, *e.g. *"Homo sapiens," "Human immunodeficiency virus" and "Human respiratory syncytial virus."

Using dictionary-based methods instead of rule-based methods, it is also possible to recognize common names, making the software more suitable for processing biomedical research articles, where authors often only refer to species by using their common (vernacular) names, such as "human" or "mouse." Recognized species names are typically normalized against the NCBI taxonomy [31]. For example, PathBinderH [14] is a dictionary-based web service where users can submit PubMed queries and filter the documents retrieved by species mentioned in the documents. Unfortunately, the service is currently limited to 20,000 species and is restricted to a fixed set of 65,000 of documents in MEDLINE. AliBaba implements a dictionary-based web service for species name recognition in PubMed abstracts and normalization to NCBI taxonomy identifiers, which includes methods to filter homonyms for common species names [32]. WhatizitOrganisms [33] is another dictionary-based system based on the NCBI species taxonomy, also available as a web service, that recognizes and normalizes species as well as other taxonomic ranks. It is a one of modules of the more general Whatizit system [33], which provides a number of different entity recognition and normalization pipelines based on dictionaries for different entity types. Neither the implementation details nor any evaluation of either AliBaba or WhatizitOrganisms system have been reported, however an analysis of WhatizitOrganisms output is presented here.

Recently, Kapeller *et al. *[10] have reported work on species name recognition and normalization in an attempt to determine the "focus organisms" discussed in a document. This system includes a dictionary-based term search combined with filters to remove common English words, and then ranks species based on their mention frequency in the abstract or main text. Evaluation is performed against a set of 621 full text documents where species mentions have been automatically generated from corresponding protein-protein interaction entries in the IntAct database [34], with a reported recall of 73.8% and precision of 74.2%. Since it is aimed at recognizing species in order to guide protein name normalization, the system is limited to the 11,444 species with entries in UniProt [35], and does not implement any disambiguation methods since levels of species name ambiguity are low in this dictionary. The software is not available either for download or as a web service.

Wang and colleagues [7,36,37] have developed a species name recognition system to aid the disambiguation and identification of other entities such as gene/protein names and protein-protein interactions. This system uses diagnostic species names prefixes along with names from the NCBI taxonomy, UniProt and custom hand-compiled dictionaries to tag species with either rule-based or machine learning techniques. This system requires other entities of interest (*e.g. *genes) to be pre-tagged as input, and only attempts to tag species mentions associated with these other entities of interest. Training and evaluation is based on two related corpora of 217 and 230 full-text documents manually annotated for proteins, genes and species. Against these evaluation sets, their rule-based approaches can achieve either very high precision (91%) with very low recall (1.6%) or intermediate values (~45%) of both performance measures [7,37]. Alternatively, their machine learning based approaches that use contextual features around entities of interest to tag species yield higher performance (~70%), but are highly biased toward species represented in the training dataset [7]. Very recently, Wang *et al. *[38] have described extensions to this system and have made their Species Word Detector method available as an UIMA component [39] together with a corpus where protein/gene mentions (but not species mentions) have been manually annotated and linked to NCBI taxonomy identifiers [40].

Finally, Aerts *et al. *[41] use a sequence-based approach to detect species referred to in biomedical text by extracting DNA sequences from articles and mapping them to genome sequences. Based on a set of 9,940 full text articles in the field of gene regulation, these authors report that the correct species can be identified (relative to the species annotated in the ORegAnno database [42]) for 92.9% of articles that contain a DNA sequence that can be mapped to a genome. No software for this approach is available as a web service or standalone application. Additionally, this approach requires that articles report a DNA sequence of sufficient length to be mapped unambiguously to a genome, which is unlikely for most abstracts and may only be available for a limited proportion of full text articles.

Here we aim to produce a robust command-line software system that can rapidly and accurately recognize species names in biomedical documents, map them to identifiers in the NCBI taxonomy, and make this software freely available for use in other text-mining and bioinformatics applications. We have named this software system LINNAEUS, in honour of the scientist who established the modern species naming conventions [22]. The goal of this work is not to discover all possible species names across publications in all domains of the life sciences, but to provide efficient methods to link species names in the biomedical literature to standard database identifiers. We perform recognition and normalization for all species names at the mention level, rather than at a document level, as document-level properties (such as focal organisms [10]) can naturally be inferred from the mention level. This also enables software built upon LINNAEUS to use the precise location of species mentions, such as in the disambiguation and normalization of other positional entities (such as genes or proteins) or in direct link-outs from mentions in semantically enhanced documents. Additionally, we aim to address which dataset is best suited for evaluating the accuracy of species name recognition software. To do so, we evaluate several automatically generated biomedical document sets with species names attached to them, and conclude that a manually annotated gold standard is necessary to reveal the true performance of species name identification systems such as LINNAEUS. We therefore also provide a new gold-standard corpus of full-text articles with manually annotated mentions of species names.

## Methods

### Overview of the LINNAEUS system

**Figure 1:**
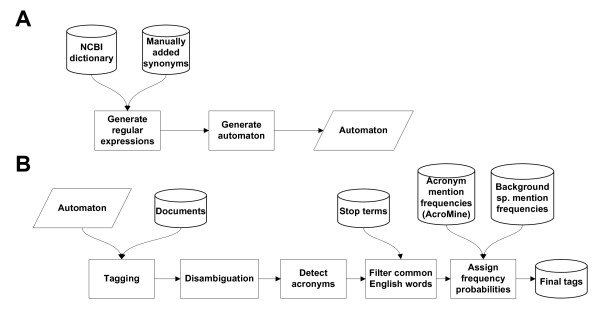
**Overview of the LINNAEUS species name identification system**. (A) Schematic diagram of the species name dictionary and automaton construction. (B) Schematic of species names tagging and post-processing.

Using the NCBI taxonomy [31] and a custom set of species synonyms, we created species dictionaries optimized for time-effective document tagging (Figure 1A). These dictionaries are used for tagging the documents, after which a number of post-processing steps are performed (Figure 1B): ambiguous mentions are disambiguated where possible using a set of heuristics, acronym definitions are detected and mentions corresponding to commonly occurring non-species terms are filtered out. Last, the species alternatives for any mentions that remain ambiguous are assigned probabilities based on their relative mention frequencies.

### Species name dictionary

The NCBI taxonomy (names data file downloaded June 1st, 2009) was used to construct the species name dictionary. This dictionary covers 386,108 species plus 116,557 genera and higher-order taxonomic units. During this work, only species were considered, but the software could easily be adapted to recognize genera or other higher-order taxonomic units as well. All species terms in the NCBI taxonomy database are categorized according to type, such as scientific name (*e.g. Drosophila melanogaster*), common name (*e.g. *fruit fly), *etc. *All types were included except for acronyms, where only a smaller subset was used (see the following section). Based on the scientific names of species, abbreviated versions of each scientific name were generated and included in the dictionary, such as "D. melanogaster" from "Drosophila melanogaster" (see also [10]). On average, each species had 1.46 names provided in the NCBI taxonomy, which rose to 2.46 names per species when abbreviations were included.

In contrast to previous work that used the UniProt species dictionary [10], substantial ambiguity is inherent in our NCBI taxonomy based dictionary, where the same term can refer to several different species. This is mostly the case with abbreviations; when abbreviated species names are not considered, the average number of species per term is 1.00088 (527,592 terms and 528,058 term-species pairs). If abbreviations are included, the number of species per term rises to 1.066 (669,578 terms, 713,525 term-species pairs).

In addition to the entries in the NCBI taxonomy, a set of additional synonyms were included that occur very frequently in the literature (see also [10,37]), such as the terms "patient," and "woman" that we assume refer to human. These could be particularly useful if no scientific names have been mentioned in a document, as often occurs in the medical literature. A full list of additional synonyms is available in Additional File 1.

Acronyms listed for species in the NCBI taxonomy are not always exact and unambiguous, in that a specific acronym can be mapped to a specific species, but in reality might be used more commonly for something else (either another species or even a non-species term). Acromine [43] is a text-mining tool that has been used to detect acronym definitions in MEDLINE, and allows users to query acronyms through a web service in order to view the declaration frequencies of that acronym. An example of an overloaded species acronym is "CMV," which in the NCBI taxonomy is mapped to "Cucumber mosaic virus." According to data generated by Acromine, CMV has been defined as "Cucumber mosaic virus" 233 times in MEDLINE, but is also much more commonly defined as "Cytomegalovirus" (7128 times). Another example is the acronym "PCV", which in the NCBI dictionary is mapped to "Peanut clump virus." In total, PCV declarations have been detected 912 times by Acromine, of which only 15 refer to different terms for "Peanut clump virus" (the most common long form is "packed cell volume," seen 490 times).

In order to overcome this problem, all acronyms listed in the NCBI taxonomy were queried against Acromine in order to retrieve frequency counts for the various expanded forms that the acronyms appear with in MEDLINE. Species recognition using LINNAEUS was then performed on the expanded-form terms in order to determine for which species each acronym was used, and their relative mention frequency (including non-species terms). The acronyms were then included in the dictionary, and the species frequencies imported from Acromine for each acronym was assigned to each potential match to the acronym. From this, it is also possible to estimate how probable it is that the acronym refers to a non-species entity. For example, the probability that PCV (mentioned above) would refer to the "Peanut clump virus" species would be 1.6% (15/912). The full list of acronyms and their associated species probabilities is available as Additional File 2.

### Automaton construction and tagging

Texts can be matched by directly using the regular expressions in the dictionary, but the large number of expressions would result in very high time requirements. Deterministic finite-state automatons (DFA) allow efficient regular expression matching, where the regular expressions for several species can be combined to greatly increase efficiency. A java implementation of DFA algorithms, the dk.brics.automaton package [44] was modified to retain species identity when joining the regular expressions of different species. Using the modified software, it is possible to find all regular expression matches (and which species they belong to) in texts in O(*n*) time, where *n *is the length of the text. Because of this implementation, the actual number of species regular expressions does not affect the time required for matching [45].

### Post-processing

After performing species name annotation using the DFA software, a number of post-processing steps are performed (see Figure 1B for overview).

#### Disambiguation

In the case of overlapping mentions of different length, the longer mention is retained and the shorter mention is removed (following the longest-match principle). This resolves cases such as "nude mice," where both the full term and the term "mice" will match (in this case to the same species), and "Human immunodeficiency virus 1" where both the full term and the shorter terms "Human immunodeficiency virus" and "Human" will match (to different species).

For mentions that remain ambiguous and where one of the possible candidate species is mentioned explicitly elsewhere in the text, all occurrences of the ambiguous term are resolved to refer to the explicitly mentioned species. This is very common, as authors often mention the full name of a species with subsequent mentions being abbreviated: for example, texts first mentioning *Caenorhabditis elegans *(an explicit mention) followed by a number of mentions of *C. elegans *(an ambiguous mention matching 41 different species) are common. If several of the candidate species are mentioned explicitly (*e.g. *both *Caenorhabditis elegans *and *Croton elegans *followed by a mention of *C. elegans*), the mention is set to refer to all the explicitly mentioned species, which (while not completely disambiguating the mention) reduces the number of potential species to which it could refer.

#### Acronym declaration detection

In addition to the acronyms annotated by LINNAEUS that are included in the dictionary, novel acronym declarations are also detected on a per document basis. When an acronym definition is detected (of the form "*species *(*acronym*)," where *species *was in the dictionary and *acronym *is a sequence of capital letters, digits or hyphens), all subsequent occurrences of that acronym are also tagged within the document.

#### Removing common English words

Based on a simple list of species names that occur commonly in the English language when not referring to the species (see Additional File 3), we remove any mention with a species-term combination in the list (see also [10,37]). This removes synonyms such as "spot" (for *Leiostomus xanthurus*) and "permit" (for *Trachinotus falcatus*), and greatly reduces the number of false positives generated by the system.

#### Assigning probabilities to ambiguous mentions

Last, any mentions that remain ambiguous are assigned probabilities of how likely that mention refers to a particular species. The probabilities for ambiguous mentions are based on the relative frequency of non-ambiguous mentions of the involved species across all of MEDLINE and the open access subset of PubMed Central full-text documents. The probabilities for acronyms are based on the relative frequencies of acronym definitions as detected by Acromine (see above). For example, for the ambiguous mention "C. elegans," the probability for *Caenorhabditis elegans *would be very high, while the probability for *Crella elegans *would be much lower. For the acronym "HIV" (which might refer to both "Human immunodeficiency virus" and, much less commonly, "the Hippocratic irrelevance variable"), the probability for it referring to "Human immunodeficiency virus" would be very high.

These probabilities enable an additional form of heuristic disambiguation: in the cases where an ambiguous mention has a species alternative with a probability higher than a given cut-off (e.g. 99%), the mention could be fully disambiguated to that species (such as for the term "C. elegans" which can be disambiguated as *Caenorhabditis elegans*). Likewise, a mention could be removed if the sum of all species-related mention probabilities is smaller than a given threshold (e.g. 1%); this can happen for acronyms where in more than 99% of cases the acronym is used for a non-species term. These levels present a trade-off between accuracy and minimization of ambiguity, and could be adjusted after tagging depending on the individual needs of the user.

### Input and output formats

LINNAEUS is capable of processing a wide range of document XML formats, including MEDLINE XML [46], PMC XML [47], Biomed Central XML [48] and Open Text Mining Interface XML [49]. In addition, it can also process plain-text documents both from locally stored files and from remote database servers. Species name recognition results can be stored to standoff-based tab-separated value files, XML documents, HTML documents (for simple visualization of results) and remote MySQL database tables.

### Document sets for species tagging

Throughout this work, three different document sets were used to recognize and normalize species names. For all sets, any documents published after 2008 were removed to create fixed and reproducible sets of documents and avoid possible discrepancies during the course of the project resulting from updates to database records.

#### MEDLINE

MEDLINE is the main database of abstracts for articles in PubMed, containing more than 18 million entries. However, many entries do not actually contain any abstract. The number of documents, if counting only entries containing an abstract published up to the end of 2008, is just over 9.9 million.

#### PubMed Central open access subset

PMC provides a set of over a million full-text articles free of charge. Unfortunately, only about 10% (105,106 published up to the end of 2008) of these are truly open access and available for unrestricted text mining. The articles in this open-access (OA) subset of PMC are referred to here as "PMC OA." The majority of the articles in PMC OA are based on XML files, but some have been created by scanning optical character recognition (OCR) of non-digital articles (29,036 documents), and a few have been created by converting portable document format (PDF) documents to text (9,287 documents). We note that for the PMC OA documents that were generated with OCR or pdf-to-text software, references are not removed from these documents. Because of this, species names occurring in reference titles may be tagged. For all other documents (MEDLINE, PMC OA abstracts and PMC OA XML documents), only the title, abstract and (if available) body text is tagged (*i.e. *reference titles are not processed).

#### Abstracts from PMC OA

The abstracts of all articles in the PMC OA set form a set referred to as "PMC OA abs." PMC OA abstracts were obtained from the abstract part of the PMC OA XML files, or from the corresponding MEDLINE entry if no such section existed in the XML file (this happens when the article has been produced through OCR or pdf-to-text tools). PMC OA abstracts consists of 88,962 documents, which notably is fewer than the number of documents in PMC OA (105,106). This is because not all PMC articles are indexed in MEDLINE, and therefore some OCR or pdf-to-text documents did not have a corresponding MEDLINE entry, making it infeasible to accurately extract the abstract. Of the 88,962 abstracts, 65,739 abstracts (74%) were extracted from XML documents, while the remainder was extracted from corresponding MEDLINE documents.

#### Division of the PMC OA full-text document set

As explained in the previous section, it is not possible to reliably extract an abstract for roughly one-fifth of all full-text articles in PubMed Central since they do not have an abstract section in the PMC XML or a corresponding MEDLINE entry. We chose not to eliminate these full-text articles from our analyses since they comprise a substantial subset of documents in PubMed Central and their exclusion may bias our results. However, their inclusion makes direct comparisons of results based on PMC OA abstracts and all PMC OA full-text documents difficult, since some documents are present in the PMC OA full-text set that are missing from the PMC OA abstract set. To solve this problem at the document level, we created the "PMC OA full (abs)" set, which contains the 88,962 full-text documents where an abstract could be extracted, allowing direct comparisons between full-text documents and abstracts on exactly the same articles. Unfortunately, this document set still does not allow direct mention-level comparisons between abstracts and full text since the offset coordinates from MEDLINE entries and PMC OA full-text documents are not compatible. Because of this, we created the "PMC OA full (xml)" set, which consists of only the 65,739 full-text documents where abstracts could be extracted from the corresponding PMC XML files. Using this PMC OA full-text XML set, it is also possible to perform mention-level comparisons on the same set of documents on the same offset coordinates. We note that "PMC OA" refers to the complete set of 105,106 full-text documents, which we alternatively denote as "PMC OA full (all)".

### Document sets for evaluation

Currently, no open access corpus of biomedical documents exists that is specifically annotated for species mentions. Thus we created a number of automatically generated evaluation sets in order to analyze the accuracy of LINNAEUS and other species name tagging software. Because of the nature of the data they are based on, many of these evaluation sets can only be analyzed at the document level. Additionally, none of these automatically generated evaluation sets were based on data specifically created in order to annotate species mentions. Because of this, we created an evaluation set of full-text articles manually annotated for species mentions. The number of documents, species and tags covered by each evaluation set is shown in Table 1 and the full set of manually annotated documents can be found at the project webpage.

**Table 1:** Species name tag sets for different evaluation corpora and LINNAEUS output

Tag set	Document set	Documents	Species	Tags
NCBI taxonomy	MEDLINE	5,237	6,871	8,701
	PMC OA abs	10	21	21
	PMC OA	12	26	26

MeSH	MEDLINE	6,817,973	824	7,388,958
	PMC OA abs	44,552	518	51,592
	PMC OA	88,826	527	57,874

Entrez gene	MEDLINE	440,084	3,125	486,791
	PMC OA abs	8,371	406	9,307
	PMC OA	9,327	428	10,294

EMBL	MEDLINE	174,074	149,598	396,853
	PMC OA abs	5,157	7,582	12,775
	PMC OA	7,374	7,867	15,136

PMC linkouts	MEDLINE	35,534	29,351	248,222
	PMC OA abs	41,054	41,070	286,998
	PMC OA	42,910	32,187	289,411

Whatizit-Organisms	MEDLINE	71,856	23,598	3,328,853
	PMC OA abs	82,410 (64,228)	25,375	3,791,412
	PMC OA	94,289	26,557	4,075,644

Manual	MEDLINE	75	176	3,205
	PMC OA abs	89 (76)	215	3,878
	PMC OA	100	233	4,259

LINNAEUS output	MEDLINE	9,919,312	57,802	30,786,517
	PMC OA abs	88,962 (65,739)	5,114	303,146
	PMC OA	105,106	18,943	4,189,681

Numbers in parentheses show the portion of abstracts that can be extracted from the document XML files, enabling mention-level accuracy comparisons (see Methods for details).

#### NCBI taxonomy citations

Some species entries in the NCBI taxonomy contain references to research articles where the species is discussed. For these documents, we assume the species are most likely mentioned somewhere in the article, allowing relative recall to be a useful measure. NCBI taxonomy citations were downloaded on June 1st, 2009.

#### Medical subject heading terms

Each article in MEDLINE has associated MeSH terms specifying which subjects are discussed in the article. A subset of these terms relates to species, and can be mapped to NCBI taxonomy species entries through the Unified Medical Language System (UMLS). However, the number of species represented by MeSH terms is limited. In total, there are MeSH terms for only 1,283 species, and only 824 species actually occur in the MeSH tags in MEDLINE. Moreover, a MeSH term given to an article is no guarantee that a term is explicitly mentioned in the document. Additionally, only a small number of the total species mentions in a document are expected to be represented in the MeSH tags (only so-called focus species), causing estimates of precision using this corpus to be less informative than recall. MeSH terms were extracted from the 2009 MEDLINE baseline distribution.

#### Entrez gene entries

Entrez gene [50] provides database entries for genes with both article references and species information. Based on these data, articles can be mapped to species. While species are often mentioned together with a gene, explicit species mentions are not guaranteed in those articles. Additionally, as the database focuses on genes rather than species, a large proportion of species mentions in this document set may not be included (for example, there will be many human mentions that do not pertain to genes, and therefore are not tagged). Thus, relative precision on the Entrez gene document set is expected to be low regardless of the real software accuracy. Entrez gene data were downloaded on June 1st, 2009.

#### EMBL records

Similarly to the Entrez gene records, many EMBL [51] sequence records also contain information about both which species the sequence was obtained from and in which article it was reported (see also [52]). This enables extraction of species-article mappings, assuming that the species is explicitly mentioned in the paper reporting the nucleotide sequence. As with the Entrez gene set, this is however not guaranteed, and any species that are discussed in addition to those with reported sequences will not be present in the evaluation set (again causing precision measures to be uninformative). Version r98 of EMBL was used for this evaluation set.

#### PubMed Central linkouts

Although not described in any publication, NCBI performs species recognition text mining on full-text articles included in PMC. These taxonomic "linkouts" can be accessed when viewing an article on PMC, and can also be downloaded through the NCBI e-utils web services. By downloading these linkouts it is possible to create an evaluation set that is relevant to both recall and precision (although only on the document level). The PMC linkout data were downloaded on June 1st, 2009.

#### WhatizitOrganisms

In order to evaluate mention-level accuracy and benchmark LINNAEUS against another species name recognition system, all documents in the PMC OA set were sent through the WhatizitOrganisms web service pipeline. Unfortunately, the Whatizit web service could not process around 10% of PMC OA documents (see Table 1), which are therefore unavailable for comparison. The WhatizitOrganisms tagging was performed June 25th, 2009.

#### Manually annotated gold-standard corpus

Because all of the previously described evaluation sets are limited by the fact that they are not specifically annotated for species names, it became clear that such a set was needed in order to measure the true accuracy of LINNAEUS. Because no such evaluation set was available, 100 full-text documents from the PMC OA document set were randomly selected and annotated for species mentions. As the focus of this work is on species rather than on genera or other higher-order taxonomic units, the corpus was only annotated for species (except for the cases where genus names were incorrectly used when referring to species).

All mentions of species terms were manually annotated and normalized to the NCBI taxonomy IDs of the intended species, except for terms where the author did not refer to the species. A commonly occurring example is "Fisher's exact test" ("Fisher" is a synonym for *Martes pennanti*, but in this case refers to Sir Ronald Aylmer Fisher, who invented the statistical test). In the cases where a species ID did not exist in the NCBI taxonomy (mostly occurring for specific species strains), they were given a species ID of 0 (which is not used in the NCBI taxonomy).

Annotated mentions were also assigned to the following categories that indicated specific features of mentions, which can be used in evaluation analyses:

(i) Lexical categories:

• Whether the term was misspelled by the author.

• Whether the author used incorrect case when spelling the species name (*e.g. *"Drosophila Melanogaster").

• Whether the term was incorrectly spelled owing to an OCR or other technical error.

(i) Syntactic categories:

• Whether the name was part of an enumeration of species names (*e.g. *in "V. vulnificus CMCP6 and YJ016," referring to two different strains of *Vibrio vulnificus*).

(iii) Semantic categories:

• Whether the author used an incorrect name (*e.g. *using genus name "Drosophila" when referring specifically to *Drosophila melanogaster *or just "Pileated" when referring to the Pileated woodpecker, *Dryocopus pileatus*).

• Whether the species term was used as an adjectival "modifier," such as in "human p53" (where the author is not actually referring to the human species, but rather a gene). Note that although the author was not referring directly to the species, these mentions are still important when extracting species mentions in order to perform, for instance, gene name recognition. We also note that while the adjective "human" in "human p53" is considered a modifier, we do not consider it a modifier in phrases such as "the p53 gene in human", where the noun "human" refers directly to the human species.

A mention may belong to several categories (for example, it may be both used as a modifier and misspelled), or not belong to any category at all (*i.e. *just being a normal mention, which is the most common case). A summary of the number of species tags associated with each category is shown in Table 2. The categories give insights into how often species names are misspelled or used incorrectly in the literature. They also enable deeper analyses of any prediction errors made by LINNAEUS or any other software evaluated against this corpus. Of the 4259 species mentions annotated in this corpus, 72% (3065) are common names, reinforcing the importance of being able to accurately identify common names when processing biomedical research articles.

**Table 2:** Composition of species tags in the manually annotated corpus and false negative predictions by LINNAEUS relative to the manually annotated corpus on the same document set

Category	Number of tags in corpus	Number of false negatives
Misspelled	46	11
Incorrect case	130	128
OCR/technical errors	18	16
Enumeration	2	1
Incorrectly used name	79	66
Modifier	1,217	125
Normal mention	2,788	12

A detailed description of the different tag categories is provided in the Methods.

In order to estimate the reliability of the manual annotations, 10% of the corpus (10 documents) was also annotated by a second annotator and the inter-annotator agreement (IAA) was calculated. In total, there were 406 species mentions annotated in the 10 documents by at least one annotator. Of these 406 mentions, 368 were annotated identically by the two annotators (both mention position and species identifier). Cohen's k measure for inter-annotator agreement [53] was calculated as k = 0.89. Details of the IAA analysis can be found in Additional File 4.

### Performance Evaluation

Tags produced by LINNAEUS were compared to those in the evaluation reference sets to determine the performance of the system. If a specific tag occurs in both the LINNAEUS set and the reference set, it is called a true positive (TP); if it occurs only in the LINNAEUS set it is called a false positive (FP); and if it occurs only in the reference set it is called a false negative (FN). This is performed both on a document level (where the location of a tag within a document is not considered) and mention level (where the tag locations have to match exactly). For the evaluation sets where information is only available on a document level, mention level evaluation is not performed. In the case of ambiguous mentions, the mention is considered a TP if the mention contains at least the "true" species (and, for mention level analyses, the location is correct). We note that LINNAEUS attempts to identify all species mentioned in a document, and thus there is no limit on the number of species reported.

## Results

We applied the LINNAEUS system to nearly 10 million MEDLINE abstracts and over 100,000 PMC OA articles that were published in 2008 or before (Table 1). Tagging of the document sets took approximately 5 hours for MEDLINE, 2.5 hours for PMC OA abstracts and 4 hours for PMC OA, utilizing four Intel Xeon 3 GHz CPU cores and 4 GB memory. (We note that the main factor influencing processing time is the Java XML document parsing rather than the actual species name tagging.) These species tagging experiments far exceed the scale of any previous report [7,10,14,23,25,36,37,41], and represent one of the first applications of text mining to the entire PMC OA corpus (see also [15,54,55]). Over 30 million species tags for over 57,000 different species were detected in MEDLINE, and over 4 million species tags for nearly 19,000 species in PMC OA. LINNAEUS identifies species in 74% of all MEDLINE articles, 72% of PMC OA abstracts, and 96% of PMC OA full-text articles. In terms of the total number of species in the NCBI taxonomy dictionary, 15% of all species in the NCBI dictionary were found by LINNAEUS in MEDLINE, 1.3% were found in PMC OA abstracts and 4.9% were found in the PMC OA full-text articles. The density of species names in MEDLINE or PMC OA abstracts is 30-fold and 3-fold lower, respectively, than that for PMC OA full-text articles; the density of species mentions is 11-fold lower in both sets of abstracts relative to full-text documents.

### Ambiguity of species mentions in MEDLINE and PubMed Central

Across all of MEDLINE and PMC OA, between 11-14% of all species mentions are ambiguous. Thus levels of species name ambiguity are on the same order as across-species ambiguity in gene names [56], and indicate that some form of disambiguation is necessary for accurate species names normalization. Levels of ambiguity for the tagged document sets before and after the disambiguation step by LINNAEUS are shown in Table 3. Ambiguity levels are calculated as the number of ambiguous mentions divided by the total number of mentions, where an ambiguous mention is counted when a mention maps to several species. The disambiguation method "none" shows values prior to any disambiguation; "earlier" disambiguates by scanning for explicit mentions earlier in the document and, for comparison, "whole" disambiguates by scanning for explicit mentions in the whole document. "Strict" disambiguation does not consider the associated probabilities of correct species mentions, whereas "approximate" represents the disambiguation of any mentions where a single species has higher than 99% probability, or the sum of all species probabilities is lower than 1%.

**Table 3:** Levels of ambiguity in LINNAEUS species tags on different document sets.

	None		Earlier		Whole	
	Strict	Approx.	Strict	Approx.	Strict	Approx.
MEDLINE	0.111	0.053	0.059	0.030	0.054	0.028
PMC OA abs	0.110	0.061	0.054	0.031	0.049	0.028
PMC OA	0.143	0.075	0.029	0.015	0.027	0.013

"None" refers to the baseline case where no disambiguation is performed, "earlier" refers to disambiguation of an ambiguous mention by searching for its explicit species mentions earlier in the document and "whole" refers to disambiguation by searching for its explicit mentions across the whole document. In the "approximate" mode, a heuristic is employed to further disambiguate ambiguous mentions based on the probability of correct species usage.

Scanning for explicit species mentions elsewhere in the text leads to roughly a two-fold reduction in ambiguity for abstracts, but nearly a five-fold reduction for full text. Approximate tagging based on probabilities of correct species usage leads to roughly a two-fold reduction in levels of ambiguity, in both abstracts and full text. Overall, less than 2.9% of mentions in full-text documents remain ambiguous when explicit mentions are sought elsewhere in the text and, combined with approximate disambiguation based on probabilities of correct species usage, levels of ambiguity drop to less than 1.5%.

### Evaluation of LINNAEUS species name tagging

Evaluation of species mentions found by LINNAEUS compared to those in the evaluation sets are shown in Table 4. For the document-level evaluation sets (NCBI taxonomy references, MeSH tags, Entrez-gene references, EMBL references and PMC linkouts), the document-level tags are compared directly against the tags found by LINNAEUS in MEDLINE, PMC OA abstracts or PMC OA documents. For the mention-level evaluation sets (WhatizitOrganisms output and the manually annotated set), tags are only compared directly between the evaluation sets and PMC OA XML, since PMC OA XML is the only document set on the same offset coordinates as the evaluation sets (see Methods). For the automatically generated sets, we interpret recall and precision in the context of how species are annotated in the evaluation set to provide a qualitative analysis of the false positives and false negatives. For the manually annotated gold standard evaluation set, a quantitative analysis of false positives and false negatives was also performed.

**Table 4:** Performance evaluation of LINNAEUS species tagging on different evaluation sets

Set	Level	Main set	TP	FP	FN	Recall	Prec.
NCBI taxonomy	Doc.	MEDLINE	6,888	10,032	(1,807)	0.7922	(0.4071)
		PMC OA abs	15	20	(6)	0.7143	(0.4286)
		PMC OA full (abs)	16	166	(3)	0.8421	(0.0791)
		PMC OA full (all)	22	196	(4)	0.8462	(0.1010)

MeSH	Doc.	MEDLINE	5,073,147	4,577,293	2,315,811	0.6866	0.5257
		PMC OA abs	36,641	49,151	(14,797)	0.7123	(0.4271)
		PMC OA full (abs)	46,484	291,872	(2,219)	0.9544	(0.1374)
		PMC OA full (all)	54,814	346,071	(2,880)	0.9201	(0.1367)

Entrez gene	Doc.	MEDLINE	346,989	171,001	(139,702)	0.7130	(0.6699)
		PMC OA abs	6,946	4,110	(2,357)	0.7466	(0.6283)
		PMC OA full (abs)	8,184	38,275	(470)	0.9457	(0.1762)
		PMC OA full (all)	9,662	42,209	(628)	0.9390	(0.1863)

EMBL	Doc.	MEDLINE	158,462	183,950	(235,745)	0.4020	(0.4627)
		PMC OA abs	4,807	4,360	(7,902)	0.3782	(0.5244)
		PMC OA full (abs)	6,601	34,447	(3,859)	0.6311	(0.1608)
		PMC OA full (all)	9,433	40,212	(5,613)	0.6269	(0.1900)

PMC linkouts	Doc.	MEDLINE	(27,259)	(23,377)	(122,596)	(0.1819)	(0.5383)
		PMC OA abs	(30,315)	(27,192)	(141,735)	(0.1762)	(0.5272)
		PMC OA full (abs)	110,288	156,012	61,656	0.6414	0.4141
		PMC OA full (all)	11,2069	163,052	61,671	0.6450	0.4073

Whatizit-Organisms	Doc.	PMC OA abs	64,686	29,222	12,930	0.8334	0.6888
		PMC OA full (abs)	308,410	67,171	100,079	0.7550	0.8211
		PMC OA full (all)	344,445	73,489	109,668	0.7585	0.8242
	
	Mention	PMC OA abs	139,077	147,426	39,351	0.7794	0.4854
		PMC OA full (xml)	1,164,799	1,596,615	527,284	0.6883	0.4218
		PMC OA full (all)	1,304,620	2,398,321	1,133,018	0.5352	0.3523

Manual	Doc.	PMC OA abs	101	0	3	0.9712	1.0
		PMC OA full (abs)	421	46	9	0.9791	0.9015
		PMC OA full (all)	462	49	9	0.9809	0.9041
	
	Mention	PMC OA abs	326	3	19	0.9449	0.9909
		PMC OA full (xml)	3,190	92	222	0.9350	0.9720
		PMC OA full (all)	3,973	120	241	0.9428	0.9707

Values in parentheses are for comparisons between document sets of different type (for example, evaluation tag sets based on full text compared against species tags generated on abstracts) or when the evaluation set is likely to exclude a large number of species mentions. PMC OA full (all) shows accuracy for all full-text documents. PMC OA full (abs) shows accuracy for all full-text documents with an abstract that can be extracted, allowing comparison of document-level accuracy between full-text and abstract. PMC OA full (xml) shows accuracy for all full-text documents with XML abstract, allowing comparison of mention-level accuracy between full-text and abstracts.

#### NCBI taxonomy citations

Results for PMC OA and PMC OA abstracts relative to the NCBI taxonomy are difficult to assess because of the low number of intersecting documents (n = 12). When comparing NCBI taxonomy citations to LINNAEUS predictions on MEDLINE, no particular species or set of terms stand out among the incorrect predictions. From an analysis of the false negatives ("missed" mentions), it seems that the majority of false negatives are not actually mentioned in the abstract, although they still could be mentioned in the main body text. The reason for the apparent low precision and high number of false positives is that the majority of species mentioned in the articles are not included in the evaluation tag set.

#### Medical subject headings

For MeSH, very few mentions are actually included in the evaluation set, as the purpose of MeSH is to identify the main themes discussed in a paper rather than each individual species mentioned. This greatly affects the number of false positives. Human stands out among the false negatives, representing 84% (1,950,767) of all false negatives in MEDLINE and 31% (1,316) in PMC OA. Inspecting a sample of documents shows that, both for human and other species, the false negatives are not explicitly mentioned in the documents. As expected, full-text documents offer higher recall relative to abstracts, since mentions located in the main body text are available to both LINNAEUS and the MeSH curators.

#### Entrez gene entries

Relative to Entrez gene, our tagging precision is low (19.0% for full-text documents) due to the fact that far from all species mentions are included in the evaluation tag set. Recall is high for full-text articles, with 93.9% of species tags in the PMC OA set correct found by LINNAEUS. Among the entries that still were missed, *Drosophila melanogaster *stands out, comprising 28.7% (184) of false negatives. Inspection shows that false negatives often appear because only the genus name "Drosophila" being used in the article as shorthand for the species *Drosophila melanogaster*, potentially warranting the addition of "Drosophila" as a synonym for *Drosophila melanogaster *(see also [10]). Among the remaining false negatives, the species seems not to be mentioned in the documents. The lower recall for abstracts relative to full text is most likely due to the species associated with a gene being mentioned in the main body text rather than in the abstract.

#### EMBL records

For the EMBL set, no species is especially over-represented among the false negatives. An inspection of the false negative samples from all three document sets reveals that the species is often not explicitly mentioned in the article. Sometimes this is because nucleotide sequences are reported in a paper for a species but only discussed in supplementary data files, which are not available to be tagged by the software. Higher recall values for full-text articles as compared to abstracts indicate that species names are more likely to be mentioned in the main body. As with the MeSH and Entrez gene document sets, precision values are of low relevance due to the evaluation set not including all species mentions.

#### PubMed Central linkouts

Performance of LINNAEUS compared to PMC linkouts reveals recall levels similar to those obtained on the EMBL document set, but lower than those for MeSH or Entrez Gene, despite the fact that this evaluation set has been constructed with the similar aim of performing species tagging as LINNAEUS (although on a document level). Inspecting a number of false positives and negatives reveals that all were incorrectly tagged in the PMC linkout evaluation set, often for no apparent reason. For some false negative cases, articles have been tagged with species whose names can only be found in the titles of the references. This suggests that species names in the PMC linkouts are detected also in referenced article titles (while in some cases linkouts are missed even when species are mentioned in the main article title). Lower performance for MEDLINE and PMC OA abstracts is due to comparing species names found by LINNAEUS only in abstracts to those found in the full documents in PMC linkouts, and as such are not directly relevant.

#### WhatizitOrganisms

The last automatically generated evaluation set we considered was from WhatizitOrganisms, which provided the opportunity to investigate the performance of LINNAEUS species tagging at both the document and mention level. LINNAEUS recall is worse at the document level when evaluated against WhatizitOrganisms relative to MeSH or Entrez Gene, but better than EMBL or PMC linkouts, while precision is higher than all the other datasets. At the mention level, relatively low values of both recall and precision of LINNAEUS tags evaluated against WhatizitOrganisms indicate substantial differences in the tagging of these two methods. When inspecting these differences, they can be seen to form three main error classes, described below.

##### Disambiguation errors

When a species term is ambiguous, WhatizitOrganisms will always return a single ID only, which can be incorrect (for instance, for all instances of "C. elegans", the ID for *Celeus elegans *is returned). In the cases where LINNAEUS has correctly disambiguated these mentions, they will result in both a false negative and a false positive relative to WhatizitOrganisms. Using the example above, the false negative would stem from *Celeus elegans *not being found, and the false positive would be caused from *Caenorhabditis elegans *being found, despite not being in the WhatizitOrganisms reference set. Most ambiguous terms (mainly abbreviations and in some cases acronyms) give rise to this kind of error.

##### Acronym errors

Acronym errors are introduced both because of ambiguities as described above (for example, "HIV" mentions are systematically tagged as *Simian-Human immunodeficiency virus *by WhatizitOrganisms), but also because some acronyms in the NCBI taxonomy have been excluded from the LINNAEUS dictionary (this will happen if Acromine has not recorded any occurrences at all of species being abbreviated for a given acronym).

##### Manual dictionary modifications

The last class consists of the terms that either are added manually as synonyms to the LINNAEUS dictionary, or are filtered out during post-processing by LINNAEUS. Common "false positive" mentions in PMC OA arise from additional synonyms including "patient" and "patients" (681,166 total) and women (120,492). Common "false negative" mentions in PMC OA arise from manually removed terms including "spot" and "spots" (32,701 total), as well as "name" and "names" (29,848 total).

#### Manually annotated corpus

To understand the true performance of the LINNAEUS system, we generated a gold standard dataset specifically annotated to evaluate species name identification software. The reliability of this gold standard is high, however some species names are likely to be omitted from this evaluation set, as shown by IAA analysis (see above). Performance of species tagging by LINNAEUS on full-text articles is very good, with 94.3% recall and 97.1% precision on mention level, and 98.1% recall and 90.4% precision on document level. Inclusion of tags from our additional synonyms such as "patient" does not explain this high level of performance alone, as we observe 91.4% recall and 96.9% precision on mention level when tags for additional synonyms are filtered out. When compared against the abstracts of the manually annotated corpus, LINNAEUS was shown to perform with 94.5% recall and 99.1% precision at the mention level, a level similar to the accuracy achieved against full-text documents. These high levels of performance for species name tagging also imply that our disambiguation methods typically identify the correct species when confronted with multiple options.

We also compared output from WhatizitOrganisms to our manually annotated corpus to understand the performance of LINNAEUS relative to another mention-level species name tagging system. Compared to our manually annotated corpus, WhatizitOrganisms achieved recall of 42.7% and precision of 66.2% on the mention level, and recall of 80.3% and precision of 69.1% on the document level. When all additional synonyms (which are not present in the WhatizitOrganisms dictionary and therefore cannot be predicted by this system) are filtered out from the evaluation set, WhatizitOrganisms achieved recall of 64.4% and precision of 66.2% on the mention level, and recall of 84.7% and precision of 69.1% on the document level. Differences in performance between the two methods arise from the discrepancies in tagging discussed in the direct evaluation between LINNAEUS and WhatizitOrganisms above. An upgraded version of WhatizitOrganisms that addresses many of these issues and shows significantly improved accuracy relative to our manually annotated corpus is due to be launched soon (Dietrich Rebholz-Schuhmann, personal communication).

Based on the categorization of manually annotated mentions, it is possible to analyze the type of false negative and false positive predictions made by LINNAEUS. False negatives are mainly due to incorrect case being used (Table 2), suggesting that an approach that ignores case might be worth exploring. False positives are more diverse: they are mostly caused by species synonyms occurring in common English, or because LINNAEUS tagged author names as species (an example is "Rice," which occurred in author names four times in the corpus). Nearly 10% of all false positives were acronyms that had been marked as probably not referring to species (the sum of mention probabilities were lower than 5%). Approximately 20% of all false positives were due to mentions being missed during manual annotation. This result is consistent with the IAA analysis, which revealed that a second curator could identify additional species tags in these documents. These omissions were not corrected during the course of evaluation in order to preserve the integrity of evaluation set. Thus, the current manually annotated corpus should be viewed as an "18 carat" gold standard, and we aim to release a "24 carat" gold standard version in the future that corrects these errors.

### Trends in species mentions

To provide an overview of commonly mentioned species in biomedical research, and to determine if our system generated interpretable results on large sets of documents, we used LINNAEUS tags to estimate the frequency of species mentions in all of MEDLINE. The ten most commonly mentioned species at the document level are shown in Table 5, and the 100 most frequently mentioned species across MEDLINE can be found in Additional File 5. This analysis counts all unambiguous mentions of a species, plus the single most likely species for ambiguous mentions. Mentions are on a document level and a single document can mention multiple species. Humans constitute the by far the most frequently discussed organism in all of MEDLINE, with almost half of all species mentions (48.4%), as has been reported previously in analyses of data used for training and testing species recognition software [7,10]. Other commonly used model organisms such as rat, mouse and baker's yeast are also represented, but somewhat more surprising is the frequent occurrence of cow, rabbit, dog and chicken. The high number of mentions for cow and rabbit are partially explained by indirect mentions of these species for their role in generating experimental reagents such as "bovine serum" or "rabbit polyclonal antibody."

**Figure 2:**
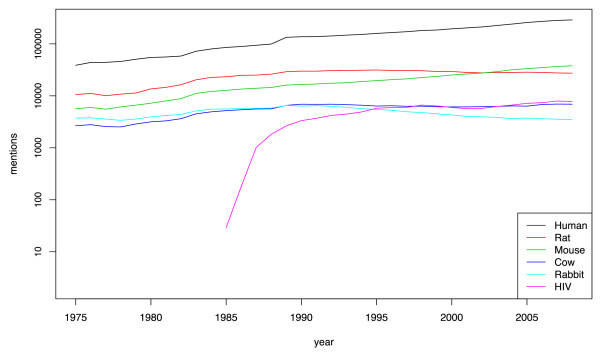
**Number of articles per year in MEDLINE mentioning human, rat, mouse, cow, rabbit and HIV since 1975**. Note that the rapid rise in mentions of the term HIV occurs just after its discovery in 1983 [59].

Utilizing species tags from MEDLINE, it is also possible to extract information about how many papers mention a species over time. Previous work on measuring trends in organism names over time has focussed on the first description of new taxa [57], while here we are interested in understanding the frequency that known species are discussed within the biomedical literature over time. Figure 2 shows document-level species mentions per year for the five most frequently mentioned species plus HIV from 1975 to the present, a timeline previously investigated for trends in gene names over time [58]. For clarity, data for the remaining species in top ten (*E. coli*, dog, baker's yeast and chicken) is not shown, but all four of these species follow the same pattern as the top five species. With the exception of HIV, all of the most frequently mentioned species have consistently been referred to at high levels over the last three decades. In contrast, the number of mentions for HIV increases rapidly after its discovery in 1983 [59]. Thus, while HIV is only the seventh most frequently mentioned species in all of MEDLINE (1975-2008) (Table 5), it is currently (2008) the fourth most frequently mentioned species after humans, mice and rats. We note that all mentions in 1985 are of the synonym "AIDS virus," since the term "Human immunodeficiency virus" was not suggested until in 1986 [60]. These results demonstrate that our species name tagging system generates meaningful predictions when applied to large sets of biomedical documents and confirm the human-centric nature of biomedical research.

**Table 5:** Top ten most commonly mentioned species in MEDLINE.

Species	Mentions	Ratio of all mentions	Ratio of all documents
Human	4,801,489	0.4743	0.4840
Rat	831,552	0.0821	0.0838
Mouse	655,695	0.0647	0.0661
Cow	186,091	0.0183	0.0187
Rabbit	162,487	0.0160	0.0163
Escherichia coli	144,077	0.0142	0.0145
HIV	117,441	0.0116	0.0118
Dog	112,366	0.0111	0.0113
Baker's yeast	112,254	0.0110	0.0113
Chicken	75,440	0.0074	0.0076

Mentions are calculated on a document level in MEDLINE relative to the total number of document-level mentions (n = 10,122,214) and the total number of documents (n = 9,919,312)

## Discussion

Species name recognition and normalization is increasingly identified as an important topic in text mining and bioinformatics, not only for the direct advantages it can provide to end-users but also for guiding other software systems. While a number of tools performing species name recognition and/or normalization of both scientific names and synonyms have been reported previously [7,10,14,23,25,33,36,37,41,61], the work presented here contributes to the field in a number of unique ways. These include availability of a robust, open-source, stand-alone application (other tools are either not publically available, only available as web services or not capable of recognizing common names), scale of species tagging (all of MEDLINE and PMC OA until 2008), depth and rigour of evaluation (other tools do not perform evaluation against normalized database identifiers, or are limited to a small sample of documents) and accuracy (compared to other available tools, LINNAEUS shows better performance, mainly due to better handling of ambiguous mentions and inclusion of additional synonyms). Moreover, we provide the first open-access, manually annotated dataset of species name annotations in biomedical text that can be used specifically to evaluate the performance of species name recognition software.

### Evaluation of species name identification software requires manually annotated gold standards

The relative performance of any bioinformatics application is only as good as the evaluation set against which it is compared. In the case of species name recognition software, no open-access manually annotated dataset of species name annotations in biomedical text existed as a gold standard for evaluation prior to the current work. During this project, we investigated four different automatically generated evaluation sets (NCBI taxonomy citations, MeSH tags, Entrez gene references, EMBL citations) based on curated document-species pairs. We also investigated two different automatically generated evaluation sets based on document-species pairs predicted using text-mining software (PMC linkouts and WhatizitOrganisms). Although it was possible to interpret the recall of LINNAEUS when the document set and the evaluation set were of the same type (*e.g. *full-text), the precision of our system could not be accurately evaluated because of incomplete or imperfect annotation of species mentions in any of these evaluation sets. We conclude that evaluation sets of document-species mappings automatically inferred from "secondary" sources such as document-gene (*e.g. *Entrez gene) or document-sequence (*e.g. *EMBL) mappings are of limited value in evaluating species name recognition software.

Because of the inherent limitations with the automatically-generated evaluation sets (including incomplete annotation of species names or incorrect disambiguation), a manually annotated evaluation corpus was created. Evaluation against the manually annotated evaluation corpus showed very good performance for LINNAEUS with 94.3% recall and 97.1% precision on a mention level, and 98.1% recall and 90.4% precision on a document level. None of the automatically generated evaluation sets come close to revealing this level of precision for species name recognition using LINNAEUS. These results underscore the importance of our manually annotated gold standard evaluation set, and suggest that evaluation of other systems on automatically generated evaluation sets (e.g. [10]) may have underestimated system precision. One interesting observation afforded by having a high quality evaluation set is that recall is higher than precision on a document level, while precision is higher than recall on a mention level. One reason for this is that when authors use non-standard or misspelled names, they will usually use those names multiple times throughout the document, leading to several false negatives on a mention level but a single only on document level. Conversely, false positives are more spread out among documents, leading to small differences in false positive counts for mention and document level evaluations.

### Improved accuracy of species name identification in full-text articles

The vast majority of text-mining research is currently conducted on abstracts of biomedical articles since they are freely available in PubMed, require fewer computational resources for their analysis, and are thought to contain the highest density of information [62,63]. Nevertheless, increasing evidence suggests that information retrieval is better on full-text articles since coverage of biomedical terms is higher relative to abstracts [62-66]. Our results for species names identification results support this conclusion, with recall of species names being higher for full-text articles relative to abstracts for the majority of evaluation sets tested (Table 4) and virtually all (96%) full-text articles being tagged with at least one species name. The benefit of performing term identification on full-text articles may be particularly useful in the case of species names, since the distribution of organism terms appears to be more uniform throughout different sections of a biomedical document than terms for diseases, genes or chemicals and drugs [62,63].

Our results also clearly demonstrate that disambiguation of species mentions by searching for explicit mentions is more successful in full-text articles than in abstracts. Thus, as has been found previously for gene names [63], the increased coverage of full-text has additional benefits for species name disambiguation, since more information is available to the disambiguation algorithms when processing full-text articles. Interestingly, we find that levels of ambiguity drop regardless of whether explicit mentions are scanned for either earlier in the text or in the whole text, possibly since the materials and methods sections of articles are often at the end of papers. After searching for explicit mentions, we find that ambiguity levels of species names in biomedical text are low (3-5%), and can be reduced even further (1-3%) using probabilistic methods if a small degree of error can be tolerated.

## Conclusions

We have developed and evaluated a robust open-source software system, LINNAEUS, which rapidly and accurately can recognize species names in biomedical documents and normalize them to identifiers in the NCBI taxonomy. The low levels of ambiguity, high recall and high precision of the LINNAEUS system make it ideally suited for automated species name recognition in biomedical text. LINNAEUS species identification in the biomedical domain could be enhanced by inclusion of names for cell lines [67], which often act as biological proxies for the species that gave rise to them. LINNAEUS is likely to also perform well in other problem domains such as the ecological and taxonomic literature provided that high quality species name dictionaries are available (e.g. [68]), although this remains an open area for future research. Further development of LINNAEUS for broader application outside the biomedical literature may require integration with other approaches such as rule-based systems for species name recognition (e.g. TaxonGrab), and we are currently aiming to provide implementations of such methods in the future that would be able to utilize the document processing methods provided by LINNAEUS. The availability of LINNAEUS now provides opportunities for downstream applications that use species names in text, including integration of species names into larger bioinformatics pipelines, semantic mark-up of species names in biomedical texts, and data mining on trends in the use of species name across documents and time.

## List of abbreviations

DFA: Deterministic finite-state automaton; IAA: Inter-annotator agreement; MeSH: Medical subject headings; NCBI: National Center for Biotechnology Information; OA: Open access; OCR: Optical character recognition; PDF: Portable document format; PMC: PubMed Central; XML: Extensible markup language

## Authors' contributions

MG developed and evaluated the software. MG and CMB conceived of and designed the project, performed the analysis, conducted the manual annotation, and drafted the manuscript. CMB and GN supervised the project. All authors revised the manuscript and approved the final manuscript.

## Supplementary Material

Additional file 1 **Additional synonyms**. A list of the synonyms that were added manually to the species dictionary. Click here for file

Additional file 2 **Added acronyms**. A list of all included acronyms and their associated species probabilities. Click here for file

Additional file 3 **Stopterms**. A list of the species/term combinations that are removed during post-processing. Click here for file

Additional file 4 **Inter-annotator agreement data**. Excel spreadsheet showing inter-annotator mentions on a species level, and the calculations used to arrive at the final inter-annotator agreement score. Click here for file

Additional file 5 **Top-100 species**. Shows the 100 most frequently mentioned species on a document level (across MEDLINE) Click here for file
